# Opinions on Computer Audition for Bowel Sounds Analysis in Intestinal Obstruction: Opportunities and Challenges From a Clinical Point of View

**DOI:** 10.3389/fmed.2021.655298

**Published:** 2021-05-28

**Authors:** Zhu Yang, Luming Huang, Jingsun Jiang, Bing Hu, Chengwei Tang, Jing Li

**Affiliations:** Department of Gastroenterology, West China Hospital, Sichuan University, Chengdu, China

**Keywords:** intestinal obstruction, bowel sounds, computer audition, machine learning, deep learning

## Introduction

Intestinal obstruction (IO) is a common acute abdominal disease with abdominal pain, distension, vomiting, and constipation. IO, especially mechanical IO (MIO), may need emergency surgery, since it possibly has high morbidity and mortality in cases of perforation, intestinal fistula, peritonitis, etc. Until now, the diagnosis of IO has still relied on abdominal imaging examination (computerized tomography, X-ray), but its application is limited by its radiation, high cost, and requirement for expensive, large equipment as well as professional technicians.

Bowel sound (BS) auscultation is not only safe and effective but also non-invasive for the diagnosis of IO. However, BS has strong subjectivity and randomness, and susceptibility to noises. Doctors have poor accuracy for BS auscultation, which was only 84.5% in normal people and even lower in patients with IO (only 70–80%) (1).

Notably, the etiology, location, and severity of all IO patients cannot be determined depending on imaging examination, symptoms and signs, and traditional auscultation of BS. As a result, intestinal ischemic necrosis or intestinal fistula may not be found in some patients until surgery, which possibly leads to delayed diagnosis and appropriate treatment with increasing the incidence of complications. Computer audition (CA), including machine learning (ML) and deep learning (DL), deals with the complex problem of understanding and analyzing sounds, such as heart sound, lung sound, and BS (2). Characteristics of BS can be automatically extracted and analyzed by powerful ML and DL. That might provide a new way to solve the above problems.

This opinion article aims to highlight the opportunities and challenges of CA for BS analysis in IO.

## Characteristics of BS

Different from heart sounds and breath sounds, there is no standard definition or classification of BS at present. This may be due to difference in duration, location, sensor of BS acquisition, and inconsistent acoustic characteristics used for classification.

In 1975, Dalle et al. used computers to analyze BS for the first time and divided BS into three types by using their duration as the classification index (3). Recently, according to duration, frequency, waveform, auditory perception, and mechanisms for the production, Du et al. classified BS as a single burst, multiple bursts, continuous random sound, harmonic sound, and a combination sound (4). When it comes to characteristics of BS in IO, there are few studies. Unfortunately, the available study showed that auscultation of BS was non-specific for diagnosing IO since there was no significant difference in sound-to-sound interval, dominant frequency, and peak frequency between patients with IO and those without IO (5). In the near future, CA may help us reveal more information about BS in IO.

## CA for BS Analysis in IO

Recently, CA has greatly facilitated BS analysis, including the following stages, acoustic sensor, the advanced digital signal processing, ML, and DL (2). DL has brought about breakthroughs in processing images, videos, and audios. The pivotal aspect of DL is that the features of signals can be learned from data using a general-purpose learning procedure. Although its application in audio recognition is still in the initial stage, it shows great advantages in terms of non-invasiveness and big data processing ability. Domestic and foreign scholars have established a DL model based on auscultation data of heart sounds and breath sounds for rapid identification of COVID-19 and its real-time and remote diagnosis (6). Unfortunately, there are few studies on DL for BS analysis in IO. Wang used back-propagation neural networks (BPNNs) for BS analysis based on spectral frequency. It was found that BPNNs may have the ability to recognize BS, with possible applications in digestive function evaluation, recovery monitoring after operation, and auxiliary diagnosis of bowel problems (7). The application of BPNNs in IO remains to be further confirmed. In addition, there is still no database of BS auscultation data for normal people and patients with IO.

## Clinical Demand for CA Of BS Analysis in The Diagnosis And Treatment Of IO

### Difficulty in Etiological Diagnosis of IO

IO can be roughly divided into three categories based on etiology, including MIO, dynamic IO, and mesenteric vascular obstruction (MVO) (8). Patients with different causes of IO are supposed to have different treatment and prognosis. Therefore, it is crucial to precisely discriminate etiologies. Usually, abdominal imaging examinations, endoscopy, and traditional BS auscultation may reveal causes of most IO cases. However, it is difficult to identify MVO and MIO in some insidious condition by using the above traditional diagnosis methods, which could easily lead to missed diagnosis or misdiagnosis. CA of BS analysis, especially ML and DL with advantages in non-invasiveness and big data processing ability, has the potential to diagnose IO of unknown etiology. Zaborski et al. demonstrated that the number of impulses of BS contributed to identify MIO caused by some tumors and diffuse peritonitis (9). Nevertheless, it is still unable to distinguish between benign and malignant tumors. Therefore, CA of BS analysis may provide a new way for etiologic diagnosis of IO.

### Difficulty in Identification of IO Location

According to the location, IO can be classified into high IO (duodenum and jejunum), low IO (small intestine), and colorectal obstruction (10). The site of IO determines the choice of internal treatment and surgical operation. However, in some cases, there are discrepancies between imaging findings and clinical conditions. So, endoscopy is needed for further diagnosis. However, application of endoscopy is not suitable for the patients with severe cardiopulmonary disease, unstable vital sign, acute cerebral accident with complications of cardiac infarction, respiratory depression, hypotension, infection, perforation, etc. Ching et al. found that multi-channel acquisition of BS could be used to identify the possible location of IO with significant difference in sound characteristics (sound duration and peak frequency) between large bowel and small bowel obstruction (5). Unfortunately, the location of IO by BS analysis is relatively rough at present with no information about specific intestinal segment provided. Therefore, there is still a long way to go for BS analysis to guide clinical work.

### Difficulty in Identification of IO Severity

In severe cases of IO, perforation, intestinal fistula, intestinal ischemia and necrosis, peritonitis, and even death may occur. They probably need emergency surgery to alleviate the condition, while mild incomplete IO can be relieved by conservative treatments. Early identification of the severity of IO may help to develop appropriate treatment and improve the prognosis of patients. In most cases, the patients can get timely surgical treatment since most complications may be recognized by imaging examination, symptoms, and signs of the patients. However, some other patients had abdominal pain relieved after medical treatment with no imaging findings of perforation, intestinal fistula, etc. Surprisingly, ischemic necrosis of the intestinal segment, and even intestinal fistula were found during surgery of those patients. Yoshino et al. used a signal processor to analyze the BS among 21 patients with MIO to evaluate the severity of IO based on the frequency and peak values of BS (11). However, there has been a lack of severity scoring systems for IO in clinical practice up to now. In relevant studies, the results could not be compared with clinical grading standards, which resulted in insufficient strength of evidence. More large sample prospective clinical studies of BS analysis may be expected to solve this problem.

### Difficulty in Monitoring of Intestinal Motility for IO Patients

Clinicians usually judge the recovery of intestinal motility in patients with MVO, dynamic IO by traditional BS auscultation, so as to guide the timing of enteral nutrition initiation. However, in clinical work, we observed that vomiting and abdominal distension occurred again after eating among some patients whose BS returned to normal. The above symptoms were relieved again after fasting with slow peristalsis and poor motility in intestinal radiography. Therefore, traditional BS auscultation cannot accurately determine the recovery of intestinal motility. In addition, repeated imaging examinations cause increased radiation exposure. Non-invasive CA of BS analysis is expected to break through this bottleneck. Spiegel used an acoustic gastrointestinal surveillance (AGIS) biosensor to identify and predict the patients at high risk of postoperative IO and to help to determine the timing of enteral nutrition initiation after surgery (12). In the near future, BS analysis is expected to judge the recovery of intestinal motility more accurately and to determine optimal timing for enteral nutrition.

## Discussion

At present, application research on CA for BS analysis in IO is relatively rare and not deep enough, and there are many aspects worthy of further improvement. ML and DL have been successfully applied in the field of acoustic-based disease diagnosis (13, 14). Since BS analysis is also one kind of acoustic-based disease diagnosis technology, we expect that the introduction of ML and DL techniques into the BS field will contribute to the research in the field.

First of all, the characteristics of BS in normal people and IO patients are still unclear. As a result, there is still no standard definition or classification of BS. We need to establish BS information database for ML and DL, analyze the characteristics of BS and unify its clinical classification and definition with the same duration, location, sensor of BS acquisition and acoustic characteristics used for classification. Secondly, in a certain situation, it is still difficult to identify the cause of IO by using only conventional diagnostic methods. We need to analyze and summarize the characteristics of BS in different causes of IO, and confirm that through clinical trials, so as to achieve etiological diagnosis of IO by using ML and DL for BS analysis. Thirdly, location accuracy for IO still needs improvement by using ML and DL to extract and analyze the characteristics of BS during IO in different intestinal segments with more specific information about IO location and sensors that can realize simultaneous auscultation of different parts of the intestine. In addition, there has been a lack of severity scoring systems for IO. Consequently, that possibly leads to the delay of treatment due to inaccurate judgment for the progress and prognosis of some patients with IO. We need to develop large sample prospective clinical trials of BS analysis by using ML and DL to promote establishment of severity scoring systems for IO. That would help clinicians to judge the severity of IO in time and effectively, and to improve the prognosis of the patients. Last but not least, we still have difficulty in judging the recovery of intestinal function in some IO patients by traditional BS auscultation, imaging examination, symptoms, and signs. The characteristics of BS should be analyzed by ML and DL in patients with different course of IO to judge the recovery of intestinal mobility more accurately and to determine optimal timing for enteral nutrition.

In conclusion, we are looking forward to making better use of ML and DL in the diagnosis and treatment of IO, so as to optimize decision-making for treatment strategy, provide precise treatment of IO and realize real-time diagnosis and monitoring of IO as soon as possible.

## Author Contributions

ZY wrote the review. JL and CT designed and revised the manuscript. ZY, LH, JJ, and BH searched and collected the literature. All authors contributed to the article and approved the submitted version.

## Conflict of Interest

The authors declare that the research was conducted in the absence of any commercial or financial relationships that could be construed as a potential conflict of interest.
